# Cu(II)/Guanidine Functionalized Disiloxane Complex of Supramolecular Structures for Visible Light-Driven Photocatalysis of Congo Red

**DOI:** 10.3390/polym14040817

**Published:** 2022-02-20

**Authors:** Maria E. Fortună, Lucia Pricop, Mirela Zaltariov, Dumitru Popovici, Maria Ignat, Valeria Harabagiu, Bogdan C. Simionescu

**Affiliations:** 1 “Petru Poni” Institute of Macromolecular Chemistry, 41A Grigore Ghica Voda Alley, 700487 Iasi, Romania; fortuna.maria@icmpp.ro (M.E.F.); pricop.lucia@icmpp.ro (L.P.); zaltariov.mirela@icmpp.ro (M.Z.); dumitru.popovici@icmpp.ro (D.P.); bcsimion@icmpp.ro (B.C.S.); 2 Faculty of Chemistry, “Alexandru Ioan Cuza” University of Iaşi, 11 Bd. Carol I, 700506 Iaşi, Romania

**Keywords:** copper(II) complex, guanidine functionalized disiloxane ligand, Congo red, visible light driven photocatalysis

## Abstract

The present study focuses on the synthesis of a new guanidine-functionalized disiloxane used as a ligand to obtain copper(II) complexes linked through hydrogen bonding into supramolecular structures. A two-step procedure was used to prepare the guanidine functionalized disiloxane ligand. Firstly, the hydrosilylation reaction between the siloxane precursor, namely 1,1,3,3-tetramethyldisiloxane (DS), and the allyl glycidyl ether (AGE) was performed in the presence of a platinum catalyst resulting in glycidoxypropyldisiloxane (DS-PMO) intermediary compound. In the second step, DS-PMO derivative was modified with 1,1,3,3-tetramethyl guanidine (TMGu) to obtain the guanidine-functionalized disiloxane ligand (bGu-DS) that was further used for the coordination of copper(II) acetate hydrate. The structures of the ligand and of its Cu(II) complex were confirmed by spectral methods (IR, UV-Vis, NMR, XRF) and correlated with theoretical calculations using semi-empirical PM6 and DFT methods. The copper(II) complex was found to exhibit low optical band gap energy (2.9 eV) and good photocatalytic activity under visible light irradiation in the decomposition of Congo Red (CR). A dye removal efficiency higher than 97% at the catalyst and CR concentrations of 1 and, respectively, 200 mg/L was obtained.

## 1. Introduction

Increasing stressing environmental concerns at the international level are related to water pollution generated by different sources of human activity. One of the most polluted wastewaters is recorded for the textile industry due to refractory dye compounds, mainly resulting from the dyeing and finishing processes. The dyes are hardly biodegradable or even non-degradable and very toxic for environmental systems. Over time, dye removal from textile effluents has been achieved by the use of chemical, physical and biological methods [1,2] and sustained research efforts are dedicated to the topic. The photodegradation of dyes in the presence of a UV and/or visible photoactive catalyst is one of the currently investigated techniques. Nowadays, researchers worldwide are fully focused on the use of heterogeneous photocatalysis for environmental remediation and wastewater treatment [3,4,5]. Usually, in heterogeneous photocatalytic processes, photon energy is used by a solid catalyst and converted into chemical energy, being really effective in the degradation of a wide range of organic contaminants. Over time, semiconductors (TiO_2_, Cu_2_O, ZnO, BiVO_4_, CdS, WO_3_ and so on) were intensively studied and applied in the photochemical degradation of various organic pollutants [6]. Recently, copper-based photosensitizers have been proved to be efficient in light-induced processes due to their great reducing power and long lifetime in an excited state [7]. Furthermore, considerable attention has been given to the visible-light photoactive copper-based photocatalysts [8,9].

Possible ligands for copper cations are guanidine derivatives. Due to their unusual donor properties, guanidines are able to coordinate transition metal ions with different oxidation states giving discrete metal complexes or extended structures. In particular, bis(guanidines) form binuclear compounds self-assembled in supramolecular coordination polymers by using different reaction conditions and/or intermolecular interactions: hydrogen bonding, π–π stacking, van der Waals forces, etc. Since 2000, a variety of copper complexes have been reported by using bis(guanindines) with ethylene and propylene backbone sequences which showed catalytic activity in atom transfer radical polymerization and oxidation catalysis. [10,11].

On the other hand, polysiloxanes are attractive catalyst supports as they are highly hydrophobic and possess excellent surface properties [12]. As a consequence of their unusual flexibility (T_g_ = −123 °C for polydimethylsiloxane), these polymers are able to expose the side chain catalytic centers to the reagents through an easy modification of their conformation. Due to their inorganic skeleton, polysiloxanes are also chemically and thermally stable (except in the presence of strong acids and bases). In addition, their organic side substituents can be easily modified [13] offering flexibility in the choice of ligand groups [14,15]. Usually, the synthesis of various classes (linear, branched, brushes, dendrimers) of polysiloxanes allows the tailoring of the structure according to the requirements of each specific process. Thus, polysiloxanes were studied either as matrices for polymer/photocatalyst composites [16] or as supports for transition metal catalysts [12,13,14,15,16,17]. For example, TiO_2_ photoactive compound was embedded into polydimethylsiloxane matrices and the resulting hydrophobic material was studied as photocatalyst in the decomposition of different dyes [18].

Due to the role transition metals played in the performance of molecular materials, remarkable catalytic properties with specific applications in photocatalysis could be obtained [19]. Among other materials, such as ZnO [20] or tridoped TiO_2_ [21], copper oxides and complexes are also known to show electronic transitions in the visible region [22,23]. Many polymers supported copper complexes for the treatment of environmental contaminants were proposed [24]. Moreover, ligand-functionalized polysiloxanes complexed with transition metals were also described [12,13,14,15,16,17], but none of them were applied in photocatalytic processes.

Considering the advantages of polymer-supported catalysts, such as their higher durability and selectivity and their easy separation from the reaction products, in this study a new guanidine modified disiloxane was synthesized. Its amphiphilic copper complex was proved to possess a polymeric supramolecular structure based on hydrogen bonding. It was expected to expose the guanidine-Cu polar moieties toward the interface between the solid complex and dye aqueous solution, and to exhibit great activity in visible light-driven photocatalytic processes. The molecular structure, morphology and physicochemical properties of the synthesized complex have been investigated by using infrared (FTIR) UV-Vis and ultraviolet-diffuse reflection (UV–DR) spectroscopies, scanning electron microscopy (SEM), differential scanning calorimetry (DSC), and thermo-gravimetric analysis (TGA), respectively. The FTIR experimental data were compared with theoretical calculations. Moreover, the synthesized copper(II) complex has been involved as a photocatalyst in the photodegradation experiment of Congo Red (CR), knowing the limited use of this anionic diazo dye in textile industry due to its high toxicity [25].

## 2. Materials and Methods

### 2.1. Materials

1,1,3,3-Tetramethyldisiloxane 97% (DS), allyl glycidyl ether 99% (AGE), Karstedt catalyst, 1,1,3,3-tetramethylguanidine 99% (TMGu), copper(II) acetate monohydrate, n-hexane, and Congo Red dye (C_32_H_22_N_6_Na_2_O_6_S_2_) (CR) were purchased from Aldrich and used as received. Toluene (Aldrich) was dried over sodium wire and distilled before use.

### 2.2. Preparation of bis(Guanidine Functionalized)-1,1,3,3-Tetramethyldisiloxane Ligand (bGu-DS)

The bGu-DS ligand was obtained in a two-step procedure previously described for the synthesis of a guanidine monofunctionalized glycidoxypropyltrisiloxane [26] (see details in Sections S1 and S2 (Supplementary Materials)). Shortly the hydrosylilation reaction of DS with AGE afforded bis(3-propoxymethyloxirane)-1,1,3,3-tetramethyldisiloxane (DS-PMO) that was further reacted with TMGu to yield the bGu-DS ligand, as a viscous oily liquid.

### 2.3. Complexation of Copper with bGu-DS

To obtain the copper complex (Cu-bGu-DS), bGu-DS (3.314 mmol) in C_2_H_5_OH (20 mL) was stirred at room temperature until complete dissolution and then Cu(CH_3_COO)_2_xH_2_O (7.57 mmol) (0.93 mmol excess) was added. After 2 h of stirring at room temperature, a fine precipitate appeared and the reaction mixture turned turquoise. The precipitate was separated from the reaction mixture by centrifugation at 3000 rpm. To the resulting deposit, 10 mL of 1/1 (*v*/*v*) ethanol/distilled water mixture were added in order to extract under stirring traces of non-reacted copper acetate. After a second centrifugation, the solid was introduced in a vacuum oven at room temperature to remove the solvent traces. A turquoise waxy material was obtained in 85% yield.

### 2.4. Photocatalytic Activity

We used 50 mg of copper(II) complex of guanidine functionalized disiloxane (Cu-bGu-DS) catalyst for all experiments. The catalyst was in contact either with 50 mL of dye solutions (solid/liquid ratio (s/l), 1/1 *w*/*v*) of different CR concentrations (50, 100 and 200 mg/L) or with 100 mL CR solution (s/l ratio, 0.5 *w*/*v*) of 100 mg/L CR concentration. The prepared mixtures containing solid catalyst and dissolved dye were stirred first in the dark for 60 min in order to achieve the adsorption equilibrium. Afterward, a low-pressure sodium lamp (400 W, producing a virtually monochromatic light averaging a 589.3 nm wavelength and a power density of 14.5 mW/cm^2^, at a distance between lamp and solution surface of 15 cm), was turned on, and the photocatalytic experiment started. The experiment lasted for 100 min, and from time to time aliquots were collected, filtered and the concentration of CR dye was checked at the specific wavelength of 498 nm by using a Shimadzu 2400 UV-Vis spectrophotometer, versus a prior registered calibration curve in the range of 0–20 mg/L CR concentrations. In order to verify whether the CR dye molecule was not affected/decomposed by the visible light irradiation used, a blind photolysis experiment was performed in the absence of the photocatalyst.

### 2.5. Measurements

Infrared spectra (FTIR) were obtained by using a Nicolet 60 SX FT-IR (Thermo Fisher Scientific, Waltham, MA, USA) under dry air, at room temperature, on KBr pellets, in the range of 4000–400 cm^−1^ or in the far IR region.

^1^H-NMR and ^13^C-NMR spectra were recorded on a Bruker Avance III 400 spectrometer (Bruker Company, Billerica, Massachusetts) in CDCl_3_.

The presence and ratio of metal and Si were evidenced using an energy-dispersive X-ray fluorescence (EDXRF) system EX-2600 X-Calibur SDD (Xenemetrix Ltd., Migdal Haemek, Israel).

Thermogravimetric data (TGA) were registered on STA 449F1 Jupiter equipment (Netzsch Company, Selb, Deutschland), in the temperature range of 30–700 °C, under airflow (50 mL/min) with a heating rate of 10 °C min^−1^.

Differential scanning calorimetry (DSC) measurements were conducted on a DSC 200 F3 Maia device (Netzsch Company, Selb, Deutschland) at heating and cooling rates of 10 °C min^−1^, under nitrogen atmosphere, at a flow rate of 50 mL min^−1^.

A Quanta 200 scanning electron microscope (FEI Company Hillsboro, OR, USA) was used to identify the surface morphology. The measurements were performed in a dry nitrogen atmosphere avoiding water absorption.

UV-Vis measurements were performed on a Shimadzu UV-2400 spectrophotometer (Shimadzu Company, Kyoto, Japan) in DMF solution. For solid state analysis an ISR-2200 integrating sphere attached to the spectrophotometer was used, allowing the registration of UV-DR spectra on MgO pellets as white standard.

## 3. Results and Discussion

A new guanidine difunctionalized disiloxane ligand, bGu-DS, was synthesized in a high yield through a two-step procedure, previously described for the preparation of a guanidine monofunctional trisiloxane [26]. The details of the synthesis and structure assessment of bGu-DS and of the intermediate product, namely bis(3-propoxymethyloxirane)-1,1,3,3-tetramethyldisiloxane (DS-PMO) are presented in Sections S1 and S2 (Supplementary Materials).

**Scheme 1:**
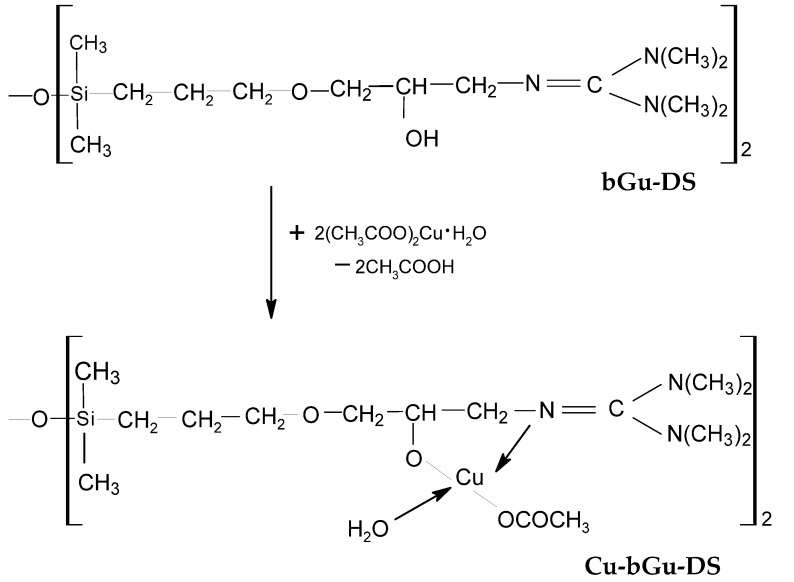
Synthesis of Cu-bGu-DS.

The as-obtained bGu-DS amphiphilic ligand was further used for the complexation of Cu(II) cations (Scheme 1), with the expectation to obtain a photocatalyst active in the visible light region. The composition and the molecular structure of Cu-bGu-DS complex were investigated by XRF, FTIR and UV-Vis spectroscopic techniques. The results obtained confirmed the theoretical calculations, while the photocatalytic efficiency of the Cu(II) complex under visible light irradiation was checked on aqueous solutions of Congo Red dye.

### 3.1. Structural Characterization of Cu-bGu-DS Complex

#### 3.1.1. X-ray Fluorescence (XRF) and Fourier Transform Infrared (FTIR) Investigation

**Figure 1:**
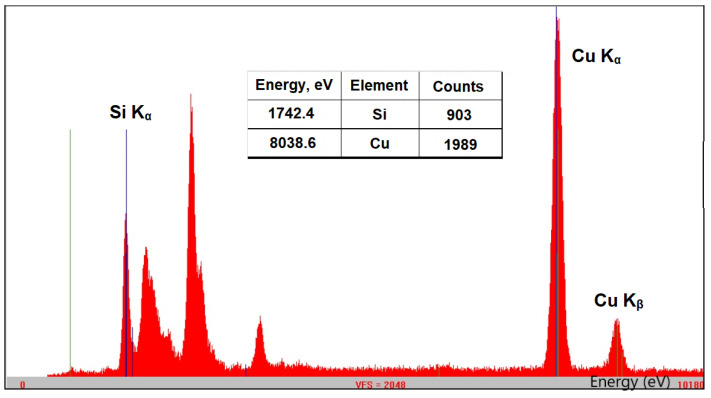
X-ray fluorescence (XRF) spectrum of Cu-bGu-DS complex showing the Kα lines associated with the transition of an electron from the L to K shell for Si and Cu atoms and the Kb of Cu (due to the transition of an electron from M to K shell); inset table: energy (eV) and intensity (counts) of Si and Cu specific transitions.

The composition and the structure of Cu-bGu-DS complex depicted in Scheme 1 were first assessed by using XRF and FTIR spectroscopies. The XRF spectrum of Cu-bGu-DS compound (Figure 1) shows the presence of both Si and Cu species. A Si/Cu atomic ratio of 1/1.03, close to the 1/1 theoretical value was calculated from XRF data with the following relation:

(1) $$\frac{Si}{Cu}=\frac{\frac{Counts_{Si}\;*\;N_{A}}{A_{Si}}}{\frac{Counts_{Cu}\;*\;N_{A}}{A_{Cu}}}$$

where, *Counts_Si_* and *Counts_Cu_* are the line intensities of *Si* and *Cu* atoms, respectively; *N_A_* is the Avogadro’s number; *A_Si_* and *A_Cu_* are the atomic masses of *Si* (28.085 a.u.) and *Cu* (63.546 a.u.), respectively.

**Figure 2:**
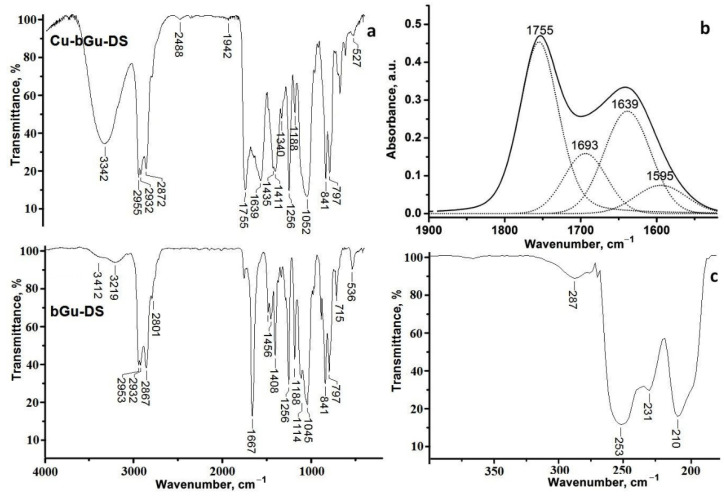
(**a**) Fourier transform infrared (FTIR) spectra of bGu-DS (**bottom**) and Cu-bGu-DS (**top**); (**b**) Deconvoluted IR spectrum of the Cu(II) complex; (**c**) far-IR spectrum of Cu-bGu-DS sample.

FTIR absorptions of bGu-DS ligand and of its copper(II) complex are depicted in Figure 2. As one may see from Figure 2a, both compounds present characteristic bands for C–H asymmetric and symmetric stretching vibrations between 2860–2960 cm^−1^, and different vibrations of methyl, methylene and C–N groups in the 1430–1475 cm^−1^ spectral interval. At 1256, 841, 797 cm^−1^ in both spectra Si-CH_3_ characteristic absorptions are visible, while the band at 1188 cm^−1^ and the large bands centered at 1052 (Figure 2a, top) or 1114–1045 cm^−1^ (Figure 2a, bottom) attributed to C–O moieties and to the superposed band of C–O–C and Si–O–Si groups, respectively, are the fingerprint of the hydroxyether-siloxane sequence.

The FTIR spectrum of the ligand (Figure 2a, bottom) reveals a large band with two maxima centered at 3219 and 3412 cm^−1^ specific to secondary hydroxyl groups linked through hydrogen bonding, while the spectrum of Cu-bGu-DS complex (Figure 2a, top) also contains the band of the crystallization water (3342 cm^−1^) and short bands assigned to hydrogen bonding between the crystallization water molecules and guanidine groups (2488 and 1942 cm^−1^) that are giving rise to a supramolecular coordination polymer [27]. The band at 1755 cm^−1^ in the spectrum of Cu-bGu-Ds complex, attributed to the C=N → Cu group, red shifted as compared to C=N band found at 1667 cm^−1^ in the bGu-DS spectrum (Figure 2a, bottom), suggests the complexation of the copper(II) cations by nitrogen atoms. Another specific band of the copper complex, attributed to the symmetric stretching vibration of the acetate group of the complex is located at 1411 cm^−1^. To find out the bands superposed in the region 1850–1450 cm^−1^, the deconvolution of the IR spectrum of Cu-bGu-DS was performed (Figure 2b). The bands at 1693 cm^−1^ and at 1639 cm^−1^ are assigned to the anion acetate group, and to the OH deformation vibrations, respectively, while the band at 1595 cm^−1^ is due to asymmetric stretching of the acetate group. The magnitude of separation of the acetate group Δ = *ν*_as_(COO^−^) − *ν*_s_(COO^−^) of the bands located at 1411 cm^−1^ (Figure 2a, top) and 1595 cm^−1^ (Figure 2b) is of 184 cm^−1^, higher than the Δ ionic (160–170 cm^−1^), proving a monodentate coordination mode of the acetate groups by Cu(II) ions [28,29], as depicted in Scheme 1.

Moreover, in the far-IR spectrum of the Cu(II) complex (Figure 2c) the presence of the absorption bands at 287, 253, 231 and 210 cm^−1^ assigned to the coordination bonds between Cu(II) and nitrogen/oxygen atoms also confirms the formation of Cu-bGu-DS complex [30].

#### 3.1.2. Theoretical Study of the Conformation of Cu-bGu-DS Complex

**Figure 3:**
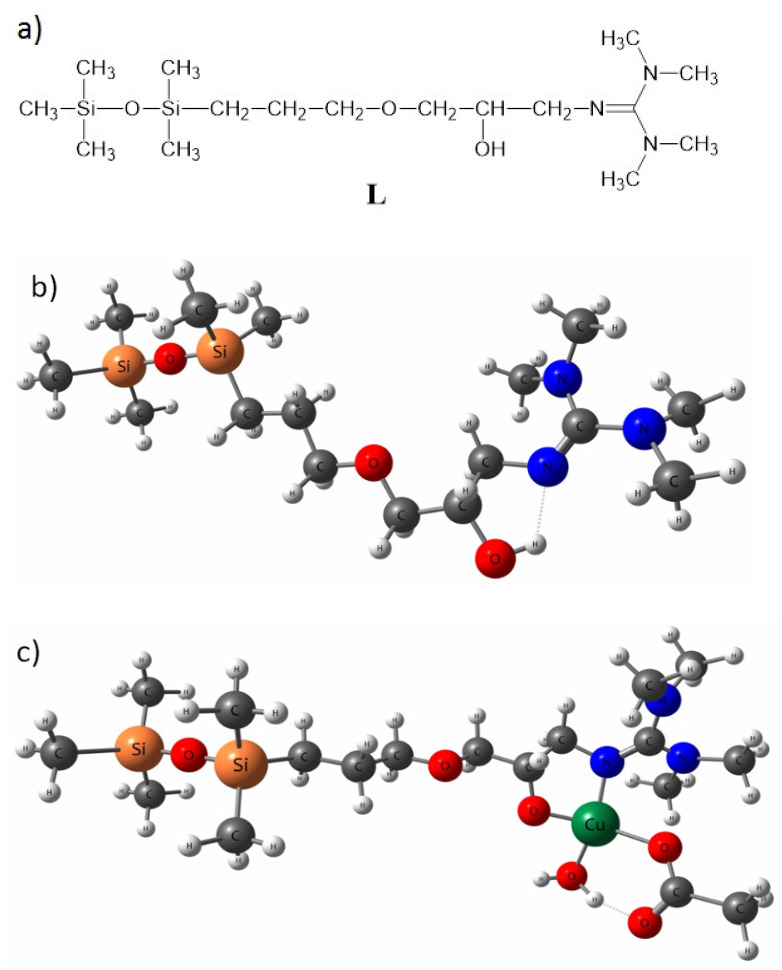
Simplified model structure (L) of bGu-DS ligand, (**a**); spatial conformation at minimum energy of L ligand, (**b**) and of Cu-L complex, (**c**).

As the bGu-DS molecule has a symmetrical structure with two ligand functionalities linked to the disiloxane group (Scheme 1), to keep the accuracy of the calculations as well as all atoms in the ligand functional group, a simplified ligand structure (L) was used for theoretical calculations. Thus, only one of the guanidine-hydroxypropoxy-ethyl ligand functionality was kept, the other one being replaced by a methyl group (Figure 3a). The spatial conformation obtained at minimum energy for L and for L-Cu^2+^ complex are shown in Figure 3b,c where the atoms are labeled as follows: white, H; dark gray, C; red, O; blue, N; orange, Si; green, Cu.

**Figure 4:**
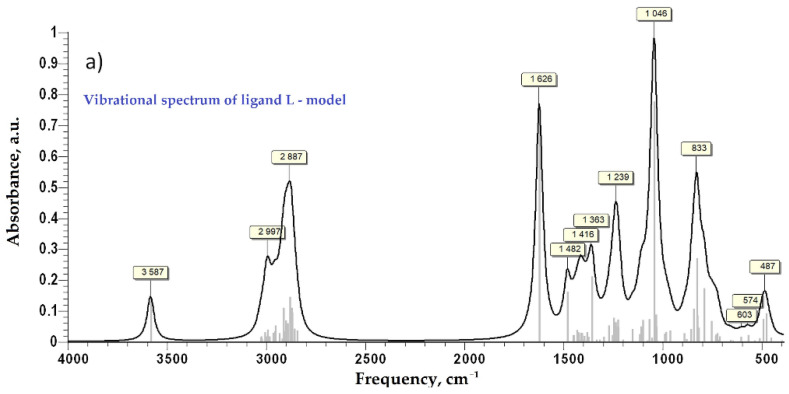

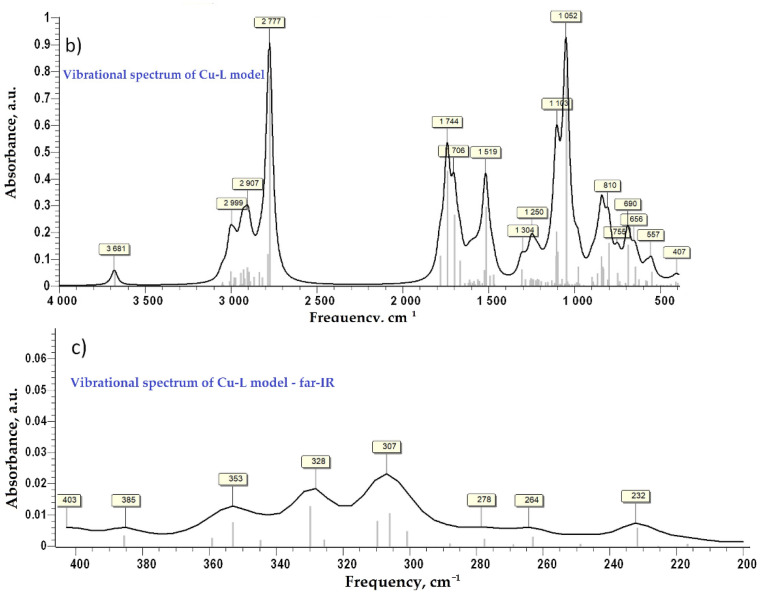
Vibrational spectra of the compound models in infra-red domain: vibrational spectrum of ligand model—L, (**a**); vibrational spectrum of Cu-L complex, (**b**); vibrational spectrum in far-infrared domain for Cu-L complex, (**c**).

After the first step of minimization using PM6 as a level of theory implemented in MOPAC2016 [31], the second step of DFT calculation at B3LYP/6-31G(d,p) as a level of theory implemented in GAMESS-US(2019 R1) under Windows was performed for each model compound [32,33,34]. No negative frequency was found at the calculation of Hessian, which indicates that the ligand and the Cu-complex are at minimum energy. The simulated vibrational spectra in the infra-red and the far infra-red regions are presented in Figure 4. For vibrational frequency lower than 2000 cm^−1^ a correction factor of 0.9627 was applied, according to the literature [35]. Close values of calculated characteristic frequency for the Cu-complex (Figure 4c) and the experimental ones (Figure 2b, bottom) were evidenced and are also compared in Table 1 for the far-IR region. Moreover, the similitude of the experimental (Figure 2a) and simulated FTIR spectra (Figure 4) is evident, proving along with XRF and FTIR findings the proposed structure of the Cu-bGu-DS monodentate complex (Scheme 1).

**Figure 5:**
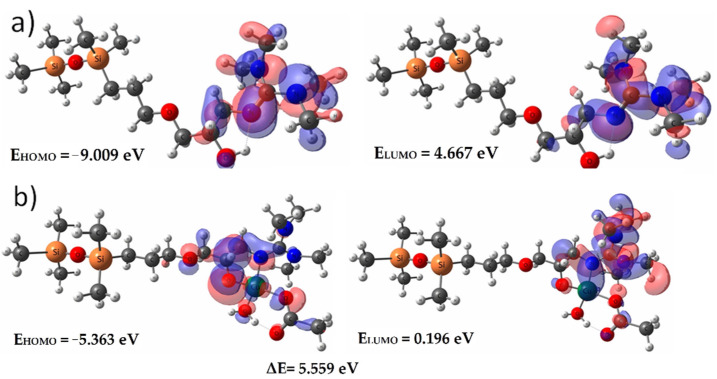
Fundamental HOMO and LUMO orbitals for (**a**) ligand model—L; (**b**) Cu-L complex.

The graphical representation of fundamental HOMO and LUMO orbitals, the energies and the band gap, for both the ligand and Cu-complex are presented in Figure 5. The presence of the metallic ion strongly disturbs the localizations and energies of these orbitals. Thus, if inside the ligand they are located on the guanidine moieties, after complexation these orbitals are shifted to the atoms that participate in the formation of the complex bonds. Moreover, the energy of these orbitals strongly increases (from E_HOMO_^L^ = −9.009 eV to E_HOMO_^Cu-L^ = −5.363 eV), while the band gap between the fundamental state of bonding and anti-bonding orbitals decreases almost 2.5 times, from ΔE^L^ = 13.676 eV to ΔE^Cu-L^ = 5.559 eV, remaining high enough to prevent electronic transitions that could generate emissions in the UV-Vis domain.

### 3.2. Thermal Behavior of the bGu-DS Ligand and of Its Cu Complex

**Figure 6:**
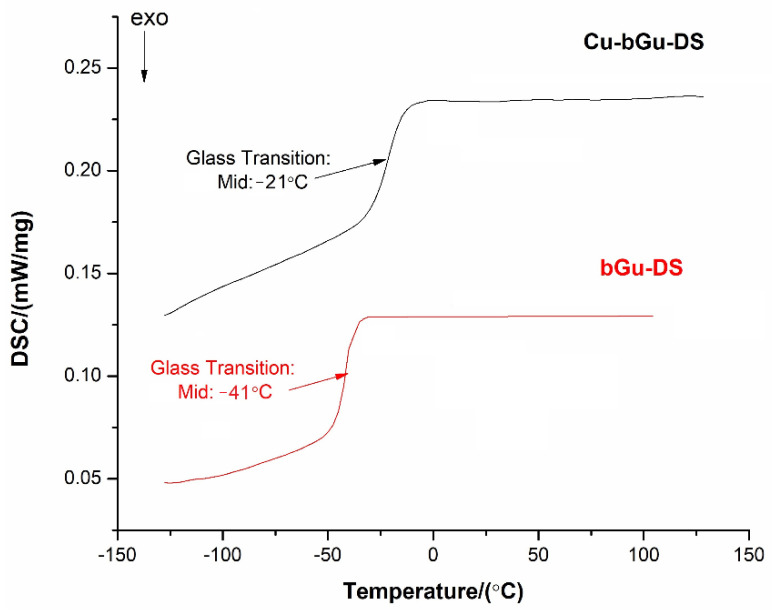
Differential scanning calorimetry (DSC) curves for the synthesized bGu-DS ligand and the Cu-bGu-DS complex.

Differential scanning calorimetry (DSC) curves (Figure 6) were registered in the temperature range between −150 and 150 °C, where only one thermal phenomenon attributed to the glass transition temperature (T_g_) was observed for each of the analyzed samples. The glass transition is a characteristic behavior of amorphous polymers, confirming the polymeric nature achieved through supramolecular physical hydrogen bonding, as evidenced by the FTIR spectrum of Cu-bGu-DS. As expected, a T_g_ value of −21 °C was found for Cu-bGu-DS complex, higher as compared to that identified for the bGu-DS precursor (about −41 °C) due to reducing molecule flexibility, following complexation. Glass transitions at low temperatures were reported also for other compounds obtained by metal coordination with ligands having highly flexible tetramethyldisiloxane sequences in their structures [36,37].

**Figure 7:**
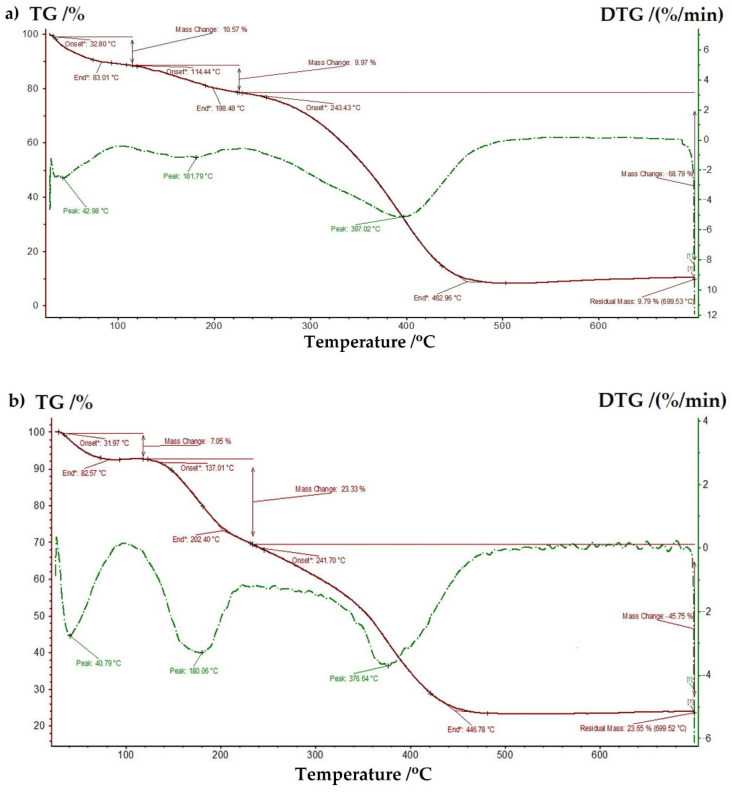
Thermogravimetric (TG) and differential thermogravimetric (DTG) curves of bGu-DS ligand (**a**) and Cu-bGu-DS complex (**b**).

Furthermore, in order to examine the thermal stability of the prepared ligand and complex, the thermogravimetric analysis was performed in the temperature range of 20–700 °C, under a nitrogen flow. The registered TG and DTG curves are presented in Figure 7. Three steps of weight loss, with maximum peaks at 43, 182, 397 °C and at 41, 180 and 377 °C were observed in TG/DTG curves of bGu-DS ligand (Figure 7a) and Cu-bGu-DS complex (Figure 7b), respectively. At low temperatures, residual solvent and adsorbed water are supposed to leave the sample, while the next two thermal phenomena are related to the loss of structural water and to the decomposition of the ligand. At temperatures ranging between 450 and 700 °C, no other decomposition phenomena were observed for both compounds up to a temperature of 700 °C. The recalculated residual masses after abstraction of the losses at the first decomposition phenomena are 10.95% for the ligand and 25.44% for its complex, close to the theoretical values of Si content in the ligand (11.65%), respectively to the sum of Si and Cu content in the complex (24.02). These results confirm once more the proposed structure for the Cu-bGu-DS complex.

### 3.3. Morphology Investigation of Cu-bGu-DS Complex

**Figure 8:**
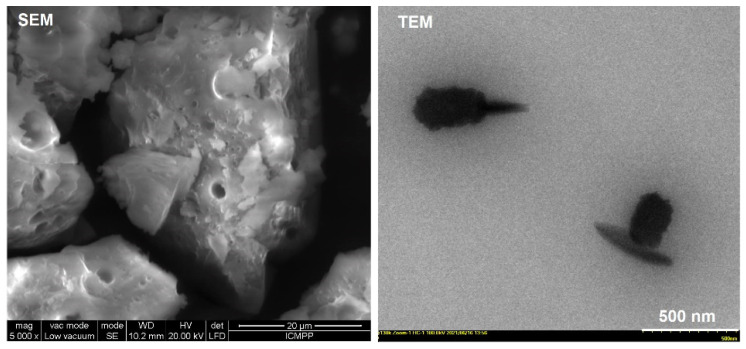
Scanning electron microscopy (SEM, **left**) and transmission electron microscopy (TEM, **right**) images of Cu-bGu-DS complex.

The prepared Cu-bGu-DS complex was investigated from morphological point of view, and SEM and TEM images are shown in Figure 8. As can be observed, the copper complex exhibits a non-uniform inner porosity, due to which a rough surface is evidenced in the SEM image, while the TEM image shows a cauliflower-like shape of several clusters of submicronic size.

### 3.4. Optical Properties of Cu-bGu-DS Complex

UV-Vis spectra of bGu-DS and of Cu-bGu-DS compounds are shown in Figure S3 (Supplementary Materials). The ligand presents one maximum at λ_max_ = 316 nm due to the n→π transitions in guanidine groups, while the Cu-bGu-DS complex reveals two maxima values at λ_max_ = 334 nm assigned to the ligand-to-metal charge transfer (LMCT) and to metal-to-ligand charge transfer (MLCT), and at λ_max_ = 688 nm attributed to d–d transitions.

**Figure 9:**
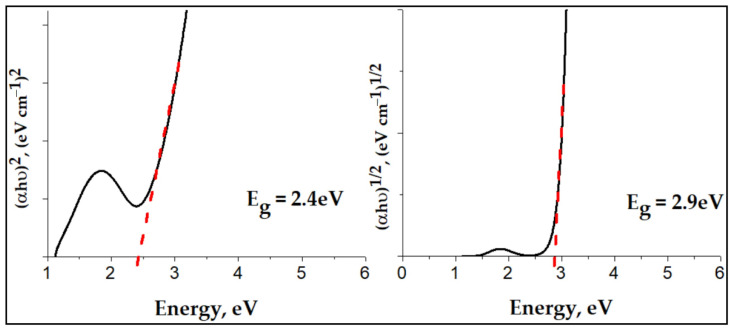
Tauc plot for indirect ( ${\left(\alpha h\vartheta \right)}^{2}$ ) and direct ( ${\left(\alpha h\vartheta \right)}^{1/2}$ ) allowed transitions of Cu-bGu-DS complex. Evaluation of the direct band gap energy.

Figure 9, displays the Tauc plots for direct and indirect allowed transitions in Cu-bGu-DS complex. The optical bandgap was calculated using the Tauc’s theory [38] ${\left(\alpha h\vartheta \right)}^{n}=K\left(h\vartheta -E_{g}\right)$ , where *α* is the absorption coefficient, *h* is Planck’s constant, *ν* is the frequency of the incident light, *K* is an energy-independent parameter, *E_g_* is the optical bandgap, and *n* = 1/2, and 2 for indirect and direct allowed transitions, respectively [39]. By plotting ${\left(\alpha h\vartheta \right)}^{1/2}$ against hϑ , corresponding to the direct allowed transitions of the Cu-complex as compared to the Tauc’s plot for indirect allowed transitions ( ${\left(\alpha h\vartheta \right)}^{2}$ against hϑ ), the best linear relationship is obtained. Thus, the direct band gap has been determined by extrapolating the straight part of the plot of ${\left(\alpha h\vartheta \right)}^{1/2}$ vs. hϑ to hϑ = 0 from the intercept of the straight line at α=0 , which is found to be 2.9 eV. With such a value of the band gap energy, the prepared Cu-bGu-DS complex seems to exhibit photocatalytic activity in the visible region. Thus, the Cu-bGu-DS complex was further subjected to photocatalytic experiments in order to evaluate its photocatalytic activity.

### 3.5. Photocatalytic Activity

**Figure 10:**
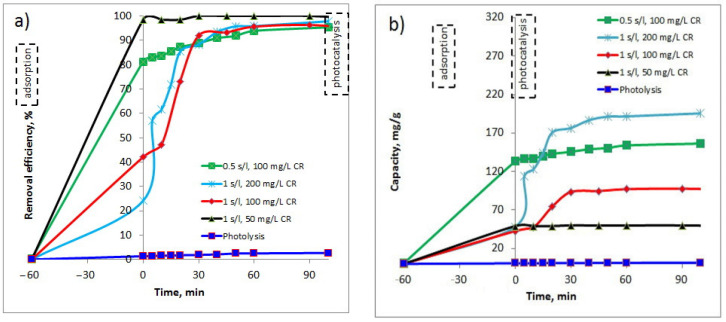
Removal efficiency (**a**) and photocatalytic capacity (**b**) of the synthesized Cu-bGu-DS complex in photodegradation of Congo Red (CR) dye.

The photocatalytic experiments envisaged the use of the synthesized Cu-complex in the degradation process of Congo Red (CR) dye, under visible light (589.3 nm). Several experiments were performed at two solid/liquid (s/l) ratios (Cu-bGu-DS photocatalyst/CR aqueous solution of 1 g/L, and 0.5 g/L) and the resulting data are shown in Figure 10. Different concentrations of CR dye solution have been considered in order to find out the optimal conditions for the photocatalytic reaction. As seen in Figure 10, Cu-bGu-DS complex, due to its hydrophobic-hydrophilic structure, is able to absorb in 60 min important amounts of CR dye, varying between 25% and 100%, depending on both the volume and the concentration of the dye solution. The maximum capacity in photodegradation of CR dye under visible light irradiation was found to be about 195 mg CR/g of Cu-complex, at a s/l ratio of 1 g/L and CR concentration of 200 mg/L (Figure 10b). As can be seen from the graph, for these experimental conditions, the photocatalyst used exhibits an adsorption capacity of CR of about 25%, allowing CR molecules to be concentrated around the Cu-complex. Afterward, when the lamp was turned on, the photodegradation of CR molecules started with the assistance of the Cu-bGu-DS complex. During the first 5 min of irradiation, the degradation efficiency rose from 25% to 58%, resulting in a fast degradation of adsorbed CR molecules on the surface of the synthesized photocatalyst. After this step of quick photodegradation, the photocatalytic process continues at a slower rate, indicating that the photocatalytic sites are further occupied by other CR molecules from solution. Thus, as the first adsorbed CR molecules are decomposed on the photocatalyst surface, other CR molecules are adsorbed on the active sites, with their almost complete removal occurring in only 50 min of the photocatalytic process. The results obtained were compared with data reported in literature which are included in Table 2. As observed from the data recorded in this table, the results obtained on Cu-bGu-DS complex compete with those obtained by Arora et al. by using Cu-loaded Fe_3_O_4_@TiO_2_ particles, when over 95% of CR in a 10 mg/L aqueous solution was photodegraded in 10 min of solar irradiation in an open space [40].

## 4. Conclusions

In this work, a new disiloxane ligand functionalized with 1,1,3,3-tetramethyl guanidine has been obtained and used to synthesize a copper(II) complex. The structure of both the ligand and its metal complex were established considering various spectral analyses (FTIR, NMR, XRF). The results obtained showed a good correlation with the theoretical calculations. Moreover, DSC investigation proved that the guanidine-functionalized disiloxane ligand and its Cu(II) complex are flexible supramolecular structures as they are characterized by negative glass transition temperatures, while their thermal stability was up to about 180 °C, as established by TG/DTG. The Cu-complex is characterized by light absorption in both UV and 500–900 nm spectral regions, and by a low value of the optical direct band gap energy (2.9 eV), demonstrating a high activity in visible light-driven catalytic photodegradation of Congo Red dye (more than 97% in 50 min). The results obtained are of great benefit for reducing the negative impact of economic activity on the environment, with applications in waste water remediation along the whole industrial chain starting from CR production and continuing with industrial sectors in which it is used, e.g., the textile industry. Further works are intended to verify the performance of siloxane-metal complexes in the photodegradation of other organic pollutants.

## Figures and Tables

**Table 1:** Experimental and calculated absorption characteristic frequencies for the Cu-complex in the far-IR region.

Experimental (cm^−1^)	Calculated (cm^−1^)	Assignment
210	216	O → Cu–O sym scissoring
231	232	O–Cu–O sym scissoring
252	263	N → Cu ← O asym scissoring
287	288	O → Cu–O sym scissoring

**Table 2:** Photocatalytic performance of the prepared Cu-bGu-DS complex in comparison with literature.

Photocatalyst	Time	Organic Pollutant	Light Wavelength	Efficiency (%)	Ref.
PDMS/TiO_2_, coating	6 days	Methylene blue (3.19 mg/L)	365 nm, natural	100%	[18]
PDMS-ZnO, powder	50 h	Phenol (1 mg/L)	410 nm, 18 W	above 95%	[20]
Mn, Mo, La/TiO_2_/AC, powder	5 h	Reactive Red 198 (20 mg/L)	Visible light Xenon lamp, 50 W	84%	[21]
Cu-loaded Fe_3_O_4_@TiO_2_ particles	10 min	Congo red (10 mg/L)	Natural solar irradiation	95%	[40]
Cu-bGu-DS complex	50 min	Congo red (200 mg/L)	589.3 nm, 400 W	>97%	This work

## Supplementary Materials

The following supporting information can be downloaded at: https://www.mdpi.com/2073-4360/14/4/817/s1, Section S1: Synthesis of bis(1,3-propyloxymethyloxirane)disiloxane (DS-PMO). Section S2: Synthesis of bis-guanidine functionalized disiloxane (bGu-DS). Section S3: UV-Vis characterisation in DMF solution. Figure S1: Schematic representation of the synthesis of DS-PMO. Figure S2: Synthesis of bGu-DS. Figure S3: UV-Vis spectra of bGu-DS ligand and Cu-bGu-DS complex in 10^−3^ M DMF solutions.
