# Supplementary material for: Cu(II)/Guanidine Functionalized Disiloxane Complex of Supramolecular Structures for Visible Light-Driven Photocatalysis of Congo Red
Source: Polymers (Basel). 2022 Feb 20;14(4):817. doi: 10.3390/polym14040817 (PMC8963006; doi:10.3390/polym14040817)
Supplement: Supplementary file 1 [file polymers-14-00817-s001.zip › polymers-1600717-supplementary.pdf]

Supporting Information – Polymers

Cu(II)/guanidine functionalized disiloxane complex of supramolecular structures for visible light driven photocatalysis of Congo Red

Maria E. Fortună[1], Lucia Pricop[1], Mirela Zaltariov[1], Dumitru Popovici[1], Maria Ignat[1,2*], Valeria Harabagiu[1*], Bogdan C. Simionescu[1]

[1]"Petru Poni" Institute of Macromolecular Chemistry,41A Grigore Ghica Voda Alley, 700487 Iasi, Romania; fortuna.maria@icmpp.ro; pricop.lucia@icmpp.ro; zaltariov.mirela@icmpp.ro; dumitru.popovici@icmpp.ro; ignat.maria@icmpp.ro; hvaleria@icmpp.ro; bcsimion@icmpp.ro
[2]"Alexandru Ioan Cuza" University of Iaşi, Faculty of Chemistry, 11 Bd. Carol I, 700506 Iaşi, Romania; maria.ignat@uaic.ro
* Correspondence: hvaleria@icmpp.ro and ignat.maria@icmpp.ro

S1. Synthesis of bis(1,3-propyloxymethyloxirane)disiloxane (DS-PMO)

DS-PMO was prepared by a hydrosilylation reaction of 0.0758 mol DS (1,1,3,3-tetramethyldisiloxane) with 0.151 mol AGE (allyl glycidyl ether), (Si–H/ AGE = 1/0.51 molar ratio), in the presence of Karsted catalyst (1mL/mol Si–H). The reaction was conducted in toluene (50% w/w) for 6 h at 70-80 °C (Scheme 1). The reaction was monitored through the disappearance of Si-H absorption band 2160 cm^{-1} in the FTIR spectrum of the reaction mixture. After the complete hydrosilylation, the reaction product was separated by vacuum distillation of the solvents and AGE in excess, and was purified by dissolution in n-hexane, filtration and vacuum evaporation of n-hexane yield, 99.5%).

^{1}H-NMR ($CDCl_3$); δ, ppm: 0.01 (Si–$\underline{C}H_3$); 0.5–0.7 (Si–CH_2); 1.3–1.7 ($CH_2C\underline{H}_2CH_2$); 2.6 and 2.8 ppm ($C\underline{H}_2$ of epoxy cycle); 3.15 ($C\underline{H}$ of epoxy cycle); 3.4–3.7 ppm ($C\underline{H}_2$–O).

^{13}C-NMR ($CDCl_3$); δ, ppm: 0.07 (Si–CH_3); 14.17 (Si–CH_2); 23.43 (CH_2-$\underline{C}H_2$-CH_2); 44.29 (CH-epoxy); 50.83 (CH_2-epoxy); 71.39 (-O-CH_2-epoxy); 74.28 (-O-CH_2).

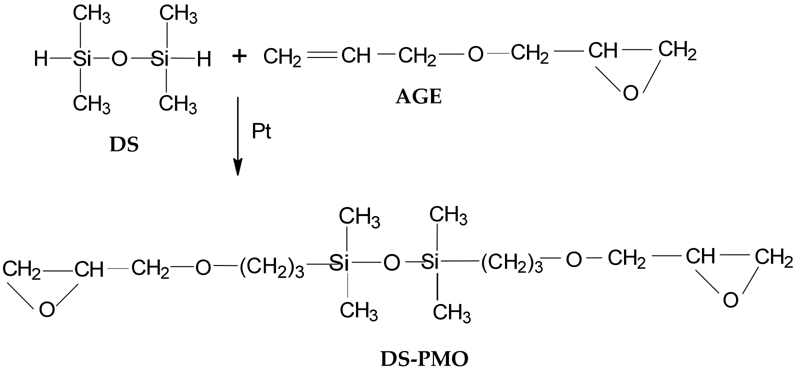

Figure S1. Schematic representation of the synthesis of DS-PMO.

S2. Synthesis of bis-guanidine functionalized disiloxane (bGu-DS)

In a typical procedure, 13.78 mmol of DS-PMO were mixed with 28.68 mmol of 1,1,3,3-tetramethylguanidine (TMGu) (1.02 mmol excess) in 25 mL toluene. The mixture was refluxed for 36 h followed by the removal of the solvent under reduced pressure to give the bifunctional bGu-DS product (yield, 98.3%).

^{1}H-NMR ($CDCl_3$); δ, ppm: 0.03-0.05 (Si–$C\underline{H}_3$); 0.45–0.52 (Si–$C\underline{H}_2$); 1.53–1.61 ($CH_2C\underline{H}_2CH_2$); 2.289 and 3.82 –3.87 (-$C\underline{H}_2$-N=); 2.92 (-N($C\underline{H}_3$)$_2$; 3.38–3.57 ($C\underline{H}_2$–O); 4.71-4.73 ppm ($C\underline{H}$-OH).

^{13}C-RMN ($CDCl_3$); δ, ppm: 0.11 (Si–$\underline{C}H_3$); 14.21 (Si–$\underline{C}H_2$); 23.38 ($CH_2\underline{C}H_2CH_2$); 37.76 (-N($\underline{C}H_3)_2$); 45.60 (-$\underline{C}H_2$-N=); 54.30 ($\underline{CH}$-OH); 72.23 (CH_2-$\underline{C}H_2$–O); 74.43 (O-$\underline{C}H_2$–CH(OH)); 162.06 (-N=$\underline{C}$).

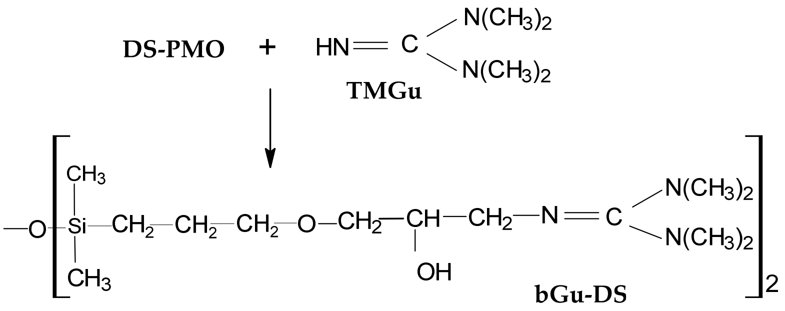

Figure S2. Synthesis of bGu-DS

S3. UV-VIS characterisation in DMF solution

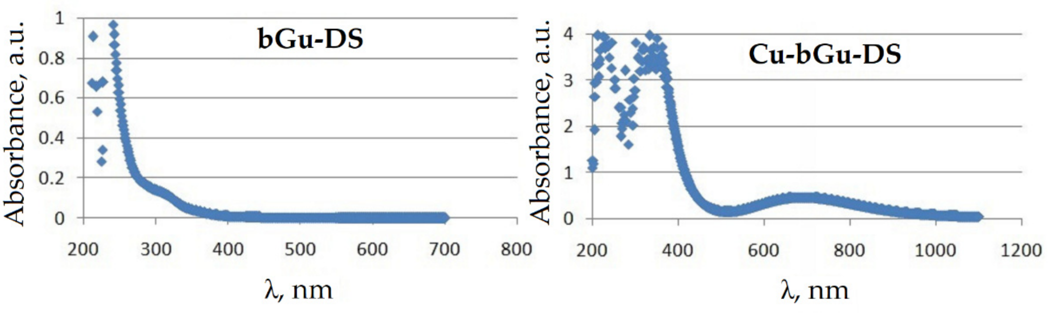

Figure S3. UV-Vis spectra of bGu-DS ligand and Cu-bGu-DS complex in 10^{-3} M DMF solutions.
